# Laparoscopic Partial Nephrectomy: Is It Worth Still Performing the Retroperitoneal Route?

**DOI:** 10.1155/2012/473457

**Published:** 2012-06-12

**Authors:** Idir Ouzaid, Evanguelos Xylinas, Géraldine Pignot, Arnaud Tardieu, Andras Hoznek, Clément-Claude Abbou, Alexandre de la Taille, Laurent Salomon

**Affiliations:** Department of Urology, Henri Mondor Hospital, APHP, Paris XII University, 94010 Créteil, France

## Abstract

*Objective*. The objective of this study was to compare perioperative, oncologic, and functional outcomes of TLPN (transperitoneal laparoscopic partial nephrectomy) versus RLPN (retroperitoneal). *Patients and Methods*. From 1997 to 2009, a retrospective study of 153 consecutive patients who underwent TLPN or RLPN for suspicious renal masses was performed. Complications, functional and oncological outcomes were compared between the 2 groups. *Results*. With a mean followup of 39 and 32 months, respectively, 66 and 87 patients had TLPN and RLPN, respectively. Tumor location was more often posterior in the RLPN and more often anterior in the TLPN. Mean operative time and mean hospital stay were longer in the TLPN group with 190 ± 85 min versus 154 ± 47 (*P* = 0.001) and 9.2 ± 6.4 days versus 6.2 ± 4.5 days (*P* < 0.05), respectively. Transfusion and urinary fistulas rates were similar in the 2 groups. After 3-year followup, chronic kidney failure occurred in 6 and and 4% (*P* = 0.67) in after TLPN and RLPN, respectively. After 3-year followup, recurrence free survival was 96.7% and 96.6% (*P* = 0.91) in the TLPN and RLPN groups, respectively. *Conclusion*. Our study confirmed that TLPN had longer operative time and hospital stay than RLPN. The complication rates were similar. Furthermore, mid-term oncological and functional outcomes were similar.

## 1. Introduction

Renal cell carcinoma (RCC) represents 2-3% of all cancers. It is the most lethal urologic cancer. Traditionally, more than 40% of patients with RCC have died of their cancer, in contrast with the 20% mortality rates associated with prostate and bladder carcinomas [1]. In 2008, there were an estimated 88 400 new cases and 39 300 kidney cancer-related deaths from RCC in Europe [2]. Etiological factors include lifestyle factors such as smoking, obesity, and hypertension. Having a first-degree relative with kidney cancer is also associated with an increased risk of RCC. The most effective prophylaxis is to avoid cigarette smoking and obesity [3]. Due to the increased detection of tumors by imaging techniques, such as ultrasound (US) and computed tomography (CT), the number of incidentally diagnosed RCCs has increased. Currently, more than 50% of RCCs are detected incidentally. These tumors are more often smaller and of lower stage [4]. Surgery is the gold standard treatment for localized RCC [3]. Nephron sparing surgery (NSS) emerged as a new surgical approach for T1 RCC. The rationale for NSS is twofold. First, it has been clearly reported that radical nephrectomy (RN) is associated with higher mortality in pT1 RCC tumors [5] and more renal failure [6]. Chronic kidney failure is independently associated with higher mortality [7]. Second, NSS has the same oncological outcomes than RN. Indications for NSS are absolute in cases with an anatomic or functional solitary kidney, relative when the functioning opposite kidney is affected by a condition that might impair renal function in the future, and elective in the presence of a healthy contralateral kidney. Another indication is patients with hereditary RCCs, who carry a high risk of developing additional kidney tumors [3]. NSS can be performed in opened (OPN), laparoscopic (LPN), or with robotic-assisted surgery. The first retroperitoneal laparoscopic partial nephrectomy (RLPN) was reported by Gill et al. in 1994 [8]. The transperitoneal laparoscopic partial nephrectomy (TLPN) was first reported by Winfield et al. in 1993 [9]. NSS can also be performed with robotic assistance [10]. Compared to OPN, LPN is associated with similar renal function outcomes, decreased postoperative narcotic use, shorter hospital stay, and improved convalescence [11]. Only few comparative TLPN and RPLN studies have been reported. The objective of our study was to compare perioperative, oncologic, and functional outcomes of TLPN versus RLPN at our institution. 

## 2. Patients and Methods

### 2.1. Patients Selection

From 1997 to 2009, 153 consecutive patients underwent TLPN or RLPN for suspicious renal masses. Data were obtained retrospectively from a prospectively maintained database. Data collection and review were approved by the Institutional Review Board. All patients underwent chest and abdominal computed tomography (CA-CT Scan) or magnetic resonance imaging (MRI). Tumor location, depth of invasion into the renal parenchyma, and relation to hilar structures were determined preoperatively. NSS was indicated by one staff surgeon in small solitary tumors (elective), bilateral and solitary kidney tumors (absolute). All procedures were performed by three high-volume surgeons (LS, ADLT, and CCA). The choice of the laparoscopic approach was at the discretion of the surgeon, and it was dictated primarily by the location and technical complexity of the renal mass. The transperitoneal approach was generally used for anterior or lateral lesions. The retroperitoneal approach was generally used for posterior, posteromedial, or posterolateral lesions. The American Society of Anesthesiologists (ASA) score was used to evaluate comorbidities. The surgical parameters, the perioperative complications, and functional and oncological outcomes were compared between TLPN and RLPN patients. Surgical complications were graded according to the Clavien score [12].

### 2.2. Surgical Technique

Our surgical technique for RLPN has been detailed previously [13–15]. Gill's technique was used to perform TLPN [16]. When the kidney was entirely dissected, a laparoscopic bull-dog clamp was introduced to ensure warm ischemia. The excision of the tumor was performed with sharp laparoscopic scissor with an adequate margin. Control of transected intrarenal blood vessels and pelvicaliceal repair were achieved with a central running stitch. Parenchymal renorrhaphy was done with sutures tied across an absorbable haemostatic bolster (Surgicel, Ethicon France 92787 Issy Les Moulineaux) or/and a biological hemostatic agent (GRF Biological Glue, Microval France, Saint-Just-Malmont, France) or Floseal (Baxter France, Maurepas, France). Tumor excision and the entire renal suture repair were performed in the ischemic kidney with the hilar vessels clamped. A perirenal drain is left for 2 days. An indwelling catheter is left for 1 day.

### 2.3. Followup

A medical examination was scheduled at one and six months and then yearly. A CA-CT Scan was performed during the followup according to the EAU Guidelines. The complications were reported with the Clavien classification system.

### 2.4. Data Collection and Statistical Analysis

Data were collected into a database, including preoperative clinical and biological characteristics, and patient demographics. Quantitative data were tested using a Student *t*-test or a Mann-Whitney test as appropriate. Qualitative data were compared using a Chi-square or a Fisher's test as appropriate. A double-sided *P* value <0.05 was considered statistically significant. The 5-year recurrence free survival was estimated according to Kaplan Meier's model, and survival curves were tested using a log-rank test. All data were analyzed using SPSS v.16.0 software (SPSS, Chicago, IL, USA).

## 3. Results

NSS was indicated electively in 84% and 91%, for solitary kidney in 11% and 6%, and for bilateral tumors in 5% and 3% in the TRLP and RLPN groups, respectively. Preoperative data are shown in Table 1. Tumor location was statistically different. In the TLPN and RLPN tumor groups, 60% and 28% (*P* < 0.001) were anterior and 13% and 49% (*P* < 0.001) were posterior, respectively. Conversely, tumor size, location pole, and the depth of the tumor in the renal parenchyma were not significatively different.

There was a longer operative time in the TLPN group (190 ± 85 min versus 154 ± 47, *P* = 0.001). The others per operative parameters are shown in Table 2. Conversion to OPN was reported in 4 patients (kidney dissection difficulties *n* = 2, peroperative bleeding *n* = 1, hilar tumor location *n* = 1) and 3 patients (kidney dissection difficulties *n* = 2, hilar control *n* = 1) in the anterior and posterior approach, respectively.

Hospital stay was 9.2 ± 6.4 days and 6.2 ± 4.5 days (*P* < 0.05) in TLPN and RLPN, respectively. We had an overall complication rate of 32% and 38% in TLPN and RLPN groups, respectively. According to the Clavien score, all grades of complications were comparable. Grade I included wound infections and positive urinalysis during the postoperative indwelling catheter period (TLPN *n* = 6 versus RLPN *n* = 7). All grade II complications were related to blood transfusion. Hematoma was drained in 1 (1.5%) and 2 (2.2%) patients and a double J stenting for urinary fistula was necessary in 3 (4.5%) and 4 (4.5%) patients in TLPN and RLPN groups, respectively. The reported grade IV complications, 3% in each group, were related to a pulmonary thrombosis (RLPN *n* = 1) and acute postoperative renal failure (TLPN *n* = 2 versus RLPN *n* = 2). Among them, one patient (1.1%) in the RLPN group required a postoperative hemodialysis. Pathological findings were similar in the 2 groups (Table 3).

**Figure 1:**
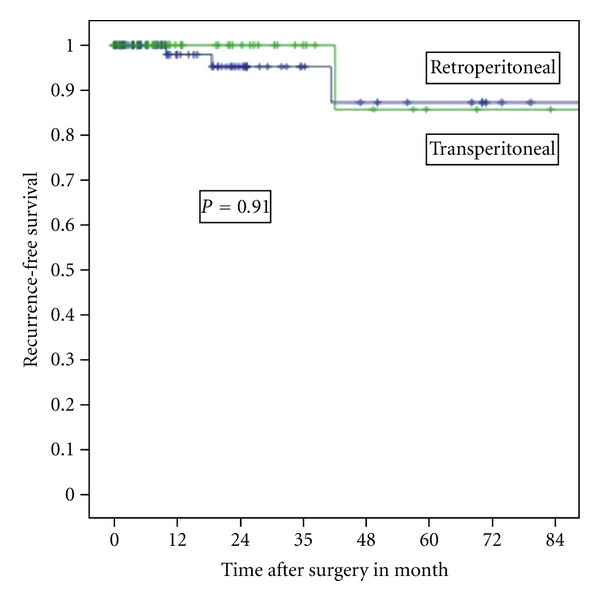
Recurrence-free survival stratified by type of surgery.

After 3-year followup, chronic kidney failure occurred in 6 and in 4% (*P* = 0.67) after TLPN and RLPN, respectively. Recurrence free-survival rates were not significatively different in the 2 groups (Table 4, Figure 1).

## 4. Discussion

 By 2000, because of the emerging data supporting elective PN, several minimally invasive surgery groups began concerted efforts to develop LPN techniques intending to closely simulate the open laparoscopic nephrectomy (OPN) procedure. Among LPN, RLPN is less popular than TLPN. Concerns about the continuing RLPN have been then arisen, and many centers stopped performing this approach. In our center, we usually use the laparoscopic retroperitoneal route for various procedures including LPN [17]. However, our staff surgeons tend to prefer the transperitoneal approach. Thus, we wanted to assess the surgical, functional, and oncologic outcomes of the two procedures.

 Regardless of the indication of the NSS, both TLPN and RLPN can be performed for absolute or elective PN. However, posterior is likely to be treated with RLPN whereas anterior tumors are treated with TLPN. Ng et al. [18] compared 100 transperitoneal and 63 retroperitoneal LPNs performed at their institution over a 3-year period. Case selection was primarily based on tumor location, with 97% of anterior tumors managed transperitoneally and 77% of posterior tumors retroperitoneally. Larger tumors and those that were deeply infiltrating were done transperitoneally regardless of location. Patients undergoing transperitoneal LPN had longer ischemia time (31 versus 28 min; *P* = 0.04), longer operative time (3.5 versus 2.9 h; *P* < 0.001), and longer hospital stay (2.9 versus 2.2 days; *P* < 0.01) compared with retroperitoneal cases. Blood loss, perioperative complications, and postoperative serum creatinine were not statistically different between groups. In concordance with Wright and Porter [19], we did not find any difference in WIT (*P* = 0.3). Furthermore, we found the same results about operative time and hospital stay in favor of the RLPN. This may be due to a rapid access to the renal helium and no mobilization of the bowl during TLPN which reduces the postoperative ileus. Our overall complication rate may seem high. However, only 3% were graded 4 and are comparable to other studies. In a thorough report on 2775 urologic laparoscopic procedures that took place at a single institution over 12 years, Permpongkosal et al. [20] reported on 345 patients who underwent LPN with an overall complication rate of 28% and a major complication rate of 5.8%. Our transfusion rate and urine leakage are comparable to Gill's et al. experience [21].

 Compared with OPN, LPN for T1A tumors had the same oncologic results. In 2004, Allaf et al. reported on 48 patients who underwent TLPN for pathologically proven renal cell carcinoma [22]. Mean tumor size was 2.4 cm, and final pathologic stage was pT1 in 88% and pT3a in 12%. No recurrences were observed in 96% of patients. Recently, Lane et al. [23] reported a long-term oncological outcome of the PN. They found that 97% and 93% patients were free of metastasis after 5 and 7-year followup. The approach for the LPN was not reported. However, the data were comparable with OPN. In previously published comparative series [18, 19, 24], the TLPN and RLPN were similar in terms of oncological outcomes. Our study confirmed these results.

 Our study is limited by the retrospective aspects of the analysis of a prospectively collected data. Besides, the study is not randomized. The choice of TLPN or RLPN is totally dependent of the surgeon's confidence and strategy. A randomized trial would give a high evidence answer to this question. Another important issue is the limited spread of RLPN. Gills et al. reported 32.6% of RLPN his first era's experience of LPN. This rate dropped to 20.8% and 1.3% in his second and third eras, respectively [21]. This may be due to the unusual anatomical presentation of the RLPN and a larger working space of the anterior approach. Recent years witnessed the development of the robotic-assisted PN [25, 26]. To date, the transperitoneal approach for the robot is mandatory. Therefore, we think the RLPN will remain unpopular. However, this approach should be considered in some patient. In fact, the risk of bowel or other intra-abdominal organ injury is dramatically reduced, and, in patients with prior intra-abdominal surgery, the need for lysis of adhesions can be avoided entirely.

## 5. Conclusion

Our study confirmed the reported data that TLPN and RLPN were similar except for the operative time and the hospital stay longer in the TLPN. However, even if the RLPN is not as popular as TLPN, this approach should be considered for few patients with past history of intraperitoneal procedures that may turn the TLPN to be more difficult than RLPN.

## Figures and Tables

**Table 1:** Baseline patients' features and tumor characteristics.

	TLPN	RLPN	*P*
No. pts	66	87	
Mean age ± SD (years)	58 ± 13	59 ±11	0.80
Sex gender (%)			
Male	57	67	0.15
Female	43	33	
Mean ASA score ± SD	1.67 ± 0.62	1.55 ± 0.64	0.74
Mean preoperative serum creatinine ± SD (*μ*mol/L)	99 ± 31	93 ± 32	0.27
Mean BMI ± SD (kg/m^2^)	24.99 ± 4.26	24.87 ± 3.92	0.75
Mean tumor size ± SD (cm)	2.64 ± 1.07	2.71 ± 1.25	0.67
Tumor location (%)			
Anterior	60	28	<0.001
Posterior	10	49	<0.001
Lateral	8	23	0.15
Tumor pole (%)			
Upper	38	22	0.18
Mid	24	37	0.38
Lower	38	41	0.72
Exophytic tumor mass (%)			
>50%	10	14	0.39
Between 25% and 50 %	53	81	0.37
Endophytic	37	5	0.07
Median nephrometry score	7.15	6.09	0.03

**Table 2:** Perioperative data and complications.

	TLPN	RLPN	*P*
No. pts	66	87	
Mean operative time ± SD (min)	190 ± 85	154 ± 47	<0.001
Mean warm ischemia time ± SD (min)	47 ± 12	25 ± 9	<0.001
Mean estimated blood loss ± SD (mL)	254 ± 187	240 ± 213	0.87
Transfusion (%)	11	16	0.45
Ureteral stenting (%)	54	41	0.32
Parenchymal reparation (%)			
Pledget	46	50	0.80
Pledget + bioadhesive agents	24	23	0.87
Bioadhesive agents	30	27	0.86
Open conversion (%)	5	5	0.90
Mean hospital stay ± SD (days)	9.2 ± 6.4	6.2 ± 4.5	<0.001
Complications (%)			
Grade 1	10	12	0.67
Grade 2	11	16	0.44
Grade 3	8	7	0.75
Grade 4	3	3	0.89
Grade 5	0	0	0.90

**Table 3:** 

	TLPN	RLPN	*P*
No. pts	66	87	
Mean followup ± SD (months)	39 ± 30	32 ± 24	0.15
Renal failure (%)	6	4	0.67
Recurrence (%)	3.1	3.4	0.91
Lost of followup	2	4	0.78

**Table 4:** 

	TLPN	RLPN	*P*
No. pts	66	87	
Malignant tumor (%)	73	70	0.45
RCC	46	43	
Papillary	27	23	
Chromophobe	0	3	
Other	0	1	
Fuhrman grade (%)			
1	15	13	0.87
2	67	64	0.78
3	18	23	0.67
Benign tumor (%)	27	30	0.77
Angiomyolipoma	14	17	
Oncocytoma	8	9	
Other	5	4	
Positive margins (%)	5	3	0.45

## References

[1] Landis SH, Murray T, Bolden S, Wingo PA (1999). Cancer statistics, 1999. *A Cancer Journal for Clinicians*.

[2] Ferlay J, Parkin DM, Steliarova-Foucher E (2010). Estimates of cancer incidence and mortality in Europe in 2008. *European Journal of Cancer*.

[3] Ljungberg B, Cowan NC, Hanbury DC (2010). EAU guidelines on renal cell carcinoma: the 2010 update. *European Urology*.

[4] Kane CJ, Mallin K, Ritchey J, Cooperberg MR, Carroll PR (2008). Renal cell cancer stage migration: analysis of the national cancer data base. *Cancer*.

[5] Thompson RH, Boorjian SA, Lohse CM (2008). Radical nephrectomy for pT1a renal masses may be associated with decreased overall survival compared with partial nephrectomy. *Journal of Urology*.

[6] Huang WC, Levey AS, Serio AM (2006). Chronic kidney disease after nephrectomy in patients with renal cortical tumours: a retrospective cohort study. *The Lancet Oncology*.

[7] Go AS, Chertow GM, Fan D, McCulloch CE, Hsu CY (2004). Chronic kidney disease and the risks of death, cardiovascular events, and hospitalization. *The New England Journal of Medicine*.

[8] Gill IS, Delworth MG, Munch LC (1994). Laparoscopic retroperitoneal partial nephrectomy. *Journal of Urology*.

[9] Winfield HN, Donovan JF, Godet AS, Clayman RV (1993). Laparoscopic partial nephrectomy: initial case report for benign disease. *Journal of Endourology*.

[10] Patel MN, Menon M, Rogers CG (2010). Robotic partial nephrectomy: a comparison to current techniques. *Urologic Oncology*.

[11] Gill IS, Matin SF, Desai MM (2003). Comparative analysis of laparoscopic versus open partial nephrectomy for renal tumors in 200 patients. *Journal of Urology*.

[12] Dindo D, Demartines N, Clavien PA (2004). Classification of surgical complications: a new proposal with evaluation in a cohort of 6336 patients and results of a survey. *Annals of Surgery*.

[13] Cicco A, Salomon L, Hoznek A (2001). Results of retroperitoneal laparoscopic radical nephrectomy. *Journal of Endourology*.

[14] Gasman D, Saint F, Barthelemy Y, Antiphon P, Chopin D, Abbou CC (1996). Retroperitoneoscopy: a laparoscopic approach for adrenal and renal surgery. *Urology*.

[15] Hoznek A, Salomon L, Antiphon P (1999). Partial nephrectomy with retroperitoneal laparoscopy. *Journal of Urology*.

[16] Haber GP, Gill IS (2006). Laparoscopic partial nephrectomy: contemporary technique and outcomes. *European Urology*.

[17] Liapis D, de la Taille A, Ploussard G (2008). Analysis of complications from 600 retroperitoneoscopic procedures of the upper urinary tract during the last 10 years. *World Journal of Urology*.

[18] Ng CS, Gill IS, Ramani AP (2005). Transperitoneal versus retroperitoneal laparoscopic partial nephrectomy: patient selection and perioperative outcomes. *Journal of Urology*.

[19] Weight JL, Porter JR (2005). Laparoscopic partial nephrectomy: comparison of transperitoneal and retroperitoneal approaches. *Journal of Urology*.

[20] Permpongkosol S, Link RE, Su LM (2007). Complications of 2,775 urological laparoscopic procedures: 1993 to 2005. *Journal of Urology*.

[21] Gill IS, Kamoi K, Aron M, Desai MM (2010). 800 Laparoscopic partial nephrectomies: a single surgeon series. *Journal of Urology*.

[22] Allaf ME, Bhayani SB, Rogers C (2004). Laparoscopic partial nephrectomy: evaluation of long-term oncological outcome. *Journal of Urology*.

[23] Lane BR, Gill IS (2010). 7-year oncological outcomes after laparoscopic and open partial nephrectomy. *Journal of Urology*.

[24] Marszalek M, Chromecki T, Mohamad Al-Ali B (2011). Laparoscopic partial nephrectomy: a matched-pair comparison of the transperitoneal versus the retroperitoneal approach. *Urology*.

[25] Gettman MT, Blute ML, Chow GK, Neururer R, Bartsch G, Peschel R (2004). Robotic-assisted laparoscopic partial nephrectomy: technique and initial clinical experience with daVinci robotic system. *Urology*.

[26] Kaul S, Laungani R, Sarle R (2007). Da Vinci-assisted robotic partial nephrectomy: technique and results at a mean of 15 months of follow-up. *European Urology*.

